# Delivering Difficult News: Simulation-Enhanced Training Improves Psychiatry Residents' Clinical Communication Skills

**DOI:** 10.3389/fpsyt.2021.649090

**Published:** 2021-03-05

**Authors:** Doron Amsalem, Andrés Martin, Mariela Mosheva, Omer Soul, Liran Korotkin, Amitai Ziv, Doron Gothelf, Raz Gross

**Affiliations:** ^1^New York State Psychiatric Institute and Department of Psychiatry, Columbia University Vagelos College of Physicians & Surgeons, New York, NY, United States; ^2^MSR–Israel Center for Medical Simulation, Sheba Medical Center, Ramat Gan, Israel; ^3^Child Study Center, Yale School of Medicine, New Haven, CT, United States; ^4^Faculty of Medicine, Tel Aviv University, Tel Aviv, Israel; ^5^Division of Child & Adolescent Psychiatry, Sheba Medical Center, Edmond and Lily Safra Children's Hospital, Ramat Gan, Israel; ^6^Sheba Medical Center, Integrated Rehabilitation Hospital, Ramat Gan, Israel; ^7^Faculty of Medicine, Sagol School of Neuroscience, Tel Aviv University, Tel Aviv, Israel; ^8^Division of Psychiatry, Sheba Medical Center, Ramat Gan, Israel

**Keywords:** simulation, residents, medical education, delivering difficult news, psychiatry

## Abstract

**Background:** Delivering difficult news to individuals diagnosed with mental health disorders and their family members can be challenging. The use of simulated patients (SP) is an effective teaching method to enhance clinical skills, particularly those around communication. We developed, implemented, and evaluated the effectiveness of an SP-based training module to improve psychiatric residents' clinical communication skills in delivering difficult news.

**Methods:** We conducted 5-h workshops consisting of 3 components: (1) a high-fidelity simulation session with a professional actor; (2) a 30-min lecture; and (3) role-playing of 3 short scenarios, during which residents rotated taking on different roles (as psychiatrist, patient, or family member). We observed through a 1-way mirror and videotaped each resident's simulation session and followed it with personalized debriefing. Following the workshop, each resident received the full-length video of their simulated interview, together with a list of questions as a take-home assignment. Two months after the workshop, the residents were invited to a second SP-based session, during which 2 independent evaluators, each a board-certified psychiatrist with expertise in medical simulation, evaluated the participants' communication skills using a previously validated instrument. To avoid observation bias, the 2 evaluators rated the videotapes blind to the timing of the simulation (pre- vs. post-training). Participants completed self-report questionnaires on satisfaction and self-confidence, before, after, and 2 months following the workshop.

**Findings:** Of the 28 psychiatric residents who participated in the training day, 24 (86%) completed the post-workshop evaluation. Mean communication score increased from 24.9 to 27.8 (paired *t*-test: 5.6, *p* < 0.001). The mean score for the self-confidence questionnaire, calculated on a 1 to 5 Likert scale, increased from 3.4 to 4.0 after the training day, and remained unchanged (4.2) 2 months later (*p* < 0.001).

**Conclusions:** An SP-based training module proved useful in improving the objectively measured communication skills of psychiatric residents delivering difficult news. The training further enhanced participants' subjective sense of confidence in those clinical skills.

## Introduction

In psychiatry, sharing diagnoses and other difficult information can be particularly challenging (1). A review of the literature shows that many psychiatrists withhold information due to various reasons (2–4). They assume patients may have restricted ability for information processing due to the nature of psychiatric symptoms, which, by their perspective, can interfere with cognitive and emotional functions as reality testing, lack of insight, and emotional regulation (5). In other studies, mental health providers reported fears about delivering an incorrect diagnosis, the patient's distress, and the stigmatizing impact of using words such as “schizophrenia” (4, 6, 7). Patients and their relatives have expressed dissatisfaction with the way diagnostic and other sensitive information was shared with them (8), and several studies have demonstrated that individuals diagnosed with mental health disorders and their relatives want to be fully informed–and should (9, 10). In sum, there is a need for clear guidelines and proper education to reduce the discomfort and uncertainty associated with sharing difficult information with individuals diagnosed with mental health disorders. In the absence of proper resources and training, psychiatrists may avoid addressing such sensitive content altogether.

Several protocols have been developed to facilitate this complicated process of information disclosure, including the SPIKES (11), and Girgis and Sanson-Fischer (12) protocols. These protocols consistently present similar principles: emotional support; what and how much information to provide; manner of communicating news; and setting (13). A study among 1,337 individuals with life-changing diagnoses showed that the SPIKES protocol largely reflects patients' preferences (14). This study found 83% of the surveyed medical schools in Canada reported using the SPIKES to teach how to disclose difficult news.

The SPIKES (11) protocol includes 6 steps: (1) *Setting* up the environment; (2) assessing the patient's *Perception* of his/her condition; (3) *Inviting* the patient to define which information he/she would like to receive; (4) providing the required *Knowledge* to the patient; (5) addressing the patient's *Emotions* with empathic responses; and (6) providing a *Summary* of treatment options and future plans. SPIKES was originally used in oncology but gradually shifted to other fields of medicine, including infectious diseases, gynecology, neurology, and ophthalmology (11, 15–18). Previous studies have identified the absence of specific guidelines for sharing difficult news in psychiatry (19) and showed the need for such protocols (20), yet the SPIKES model has not yet been empirically studied in psychiatry (21).

Delivering the diagnosis of a serious illness is an important skill in all fields of medicine, and residents consistently identify a desire and need for further education (22–24). Among various training approaches, the use of simulated patients (SPs) offers numerous advantages in medical education, which have been well-reviewed in the literature (25–27). Practicing specific skills in a supportive space without risk of harm to patients by someone inexperienced, allows for educators and trainees to focus their attention on the complexities of the skills at hand, such as delivering difficult news (28, 29). However, while SP-based methodology is widely used in medical education, its use in the psychiatric field has emerged only recently (30–32). In psychiatry, as in other fields, assisting residents to acquire skills in delivering diagnosis is pivotal (33, 34). Similar to oncology and palliative care, there are complexities and potential harm that can emerge from poorly shared information, yet in psychiatry, the use of simulation to practice communication skills has been minimally studied.

Most studies on SP-based training in delivering difficult news in medicine have focused on self-efficacy and satisfaction outcomes to address communication skills, providing findings that are solely based on the learner's subjective perceptions (35). There are only a few studies that have attempted to examine objective changes in learner performance through the use of SPs and external evaluators (36–38): a study of 38 residents in family medicine and internal medicine (37); a study of 34 residents in pediatric emergency medicine (38); and a study of 98 residents, mainly in surgery-related specialties (36). However, such an approach is yet to be undertaken in psychiatry.

We developed an SP-based workshop to enhance psychiatric residents' clinical and communication skills in delivering difficult news to patients and their families. We hypothesized that following participation in the workshop, psychiatry residents would improve their abilities to communicate difficult news as demonstrated by measurable increases in: (1) objective performance as assessed by external evaluators, and (2) self-confidence.

## Materials and Methods

### Subjects and Ethics Approval

Participants were psychiatry residents recruited from 3 free-standing psychiatric hospitals and one general hospital. All training sites were located in the center of Israel and academically affiliated with Tel-Aviv University. The Sheba Medical Center Institutional Review Board approved the study (Protocol #SMC5912-19). All participants signed an informed consent form. The study was conducted between March 2019 and January 2020.

### Workshop and Procedure

In partnership with the Israel Center for Medical Simulation (MSR), Tel Aviv University, and Yale School of Medicine, we developed a 5-h workshop designed to teach psychiatry residents how to communicate difficult information to patients or their family members. During the course of the study, we delivered the workshop 5 times, with a maximum of 6 psychiatric residents each time, in order to provide each participant with the opportunity to interact with an SP. For consistency, the same SPs attended all the workshops and played the same role each time. The workshop consisted of 3 components: (1) *High-fidelity simulation:* each participant had a 15-min interaction with an SP depicting one of two scenarios described below, followed by a small-group debriefing session. Video cameras recorded the encounters for further analysis, feedback, and reflection in the debriefing sessions. Each participant encountered one SP and then observed other participants through a 1-way mirror. Debriefings were led by two psychiatrists (DA and RG) with expertise in video-based debriefing, who highlighted core elements of delivering difficult news and communication skills. All participants were encouraged to reflect and share their feelings and thoughts, and to make suggestions for improvement; (2) *SPIKES protocol:* a 60-min presentation focusing on aspects unique to psychiatry. The SPIKES tool was chosen following a literature review because of its wide use, simplicity, and applicability for delivering difficult information to patients and their relatives (11, 39). Participants were invited to discuss their own issues and concerns regarding the delivery of difficult information; and (3) *Role-playing:* a 90-min session with 3 short scenarios, during each of which 2 residents rotated taking on different roles (as psychiatrist, patient, or family member). At the end of the training day, each resident received the full-length video of their simulated interview, together with a list of questions as a take-home assignment. Two months after the training day, each resident was invited to a second videotaped SP-based session.

### Scenarios

We created two scenarios for SPs, each based on different psychiatric vignettes of a patient and a family member. The first scenario describes a discussion with the mother of an 18-year-old patient recently admitted to a locked inpatient unit due to a first psychotic episode. The second scenario describes a hospitalized patient with bipolar disorder being recommended long-term use of lithium.

Scenarios for role plays included 3 different discussions between a psychiatrist and a patient or family member. For each scenario, we provided 2 summary cards: one for the resident who role played the psychiatrist, another for the one who played the patient or family member. During each conversation, the practicing residents rotated between roles to experience the entire spectrum of the interaction. The first scenario describes a mother being given an autism diagnosis for her 3-year-old son. The second scenario describes a 36-year-old woman with schizoaffective disorder who during an outpatient visit updates her psychiatrist on her efforts to conceive, while on treatment with valproate, a potentially teratogenic medication. The third scenario describes a 43-year-old man diagnosed with bipolar disorder being informed that his driver's license will be suspended following a recent psychotic episode.

### Evaluation

Two independent evaluators (MM and OS), each a board-certified psychiatrist with expertise in medical simulation, evaluated the participants' communication skills. To avoid observation bias, the 2 evaluators rated the videotapes blind to the timing of the simulation (pre-workshop vs. two-month post-workshop). The communication tool was developed by Kurtz et al. (40) and was previously used (41) to evaluate residents' communications skills. The communication tool is based on the SPIKES protocol and includes 17 items, such as “Introduces themselves and their roles” to examine the *Setting* step, or “Assesses the patient's starting point” to assess the *Perception* step. Response options were: 0 = “not done or inadequate,” 1 = “adequate,” and 2 = “good.” The scale's Cronbach's alpha was 0.93 for this study; inter-rater agreement was moderate (kappa = 0.58, *p* < 0.001).

Participants completed self-report questionnaires on their confidence in delivering difficult news before, after, and 2 months following the workshop. The questionnaire was previously used by Tobler et al. (41) and included 13 items such as “I feel confident in my ability to listen to patients' concerns,” or “I feel confident in my ability to summarize information in a way that is easy to understand.” Response choices ranged from 1 (“strongly disagree”) to 5 (“strongly agree”) on a Likert scale. The questionnaire was translated into Hebrew and then back-translated to English by 2 bilingual authors (DA and LK). The 2 translators and a third author (DG) discussed and resolved discrepancies in the translation. The scale's Cronbach alpha for this study was 0.81. In addition, a 6-item questionnaire was used to measure the participant's self-efficacy and satisfaction at the end of the workshop. Response choices ranged from 1 (“not at all”) to 5 (“very much”) on a Likert scale. The scale's Cronbach alpha was 0.92 for this study.

### Statistical Analysis

We calculated mean scores and standard deviations for each of the questionnaires. We used the *Shapiro Wilk*-test to evaluate for normal distribution. We then used paired *t*-tests to compare change in communication skills mean scores before and 2 months after the workshop. We also used paired *t*-tests to compare change in resident self-assessment mean scores between pre- and post-workshop time points, and between pre- and 2-months post-workshop. We used a two-tailed *p*-value of 0.05 as threshold for significance. We conducted all statistical analyses using IBM SPSS software, version 26.0 (Armonk, NY).

## Results

### Sample Characteristics

Our study sample consisted of 28 psychiatric residents (17 females, 11 males) who participated in the study and completed the pre- and post-workshop evaluations, 24 (86%) of whom also completed the two-month post-workshop evaluation. Participants' age distribution was as follows: under 25 (*n* = 2, 7%); 25 to 30 (*n* = 11, 39%); and 30 to 35 (*n* = 15, 54%). Distribution according to years in residency training was as follows: under 2 years (*n* = 16, 57%); more than 2 years (*n* = 12; 43%). Across all time points, independent *t*-tests showed no differences in total mean scores between females and males, or between residents in general or psychiatric hospitals, or between more- and less-experienced residents.

### Evaluators' Assessment of the Residents' Performance

In Table 1 we summarize mean scores for the 17-item communication skills assessment, as rated by the 2 independent evaluators blind to time period (pre- or 2 months post-workshop). Mean scores increased from 24.9 to 27.8 (*t* = 5.55, *p* < 0.001) between pre- and 2-month post-workshop, reflecting improvement in residents' performance. We also found improvement from pre- to 2-month post-workshop in 5 of the 17 items.

**Table 1:** Evaluators' scores on communication skills, blinded to the time period (before the workshop and 2 months after).

	**Pre-workshop**	**2 months** **Post-workshop**	**Statistic**	
**SPIKES component/communication skill**	**Mean (SD)**	**Mean (SD)**	***t***	***p***
**Setting**				
Greets the patient and obtains his/her name and use it	1.6 (0.5)	1.8 (0.4)	2.0	NS
Introduces themselves and their role	1.3 (0.7)	1.5 (0.6)	1.3	NS
Explains the nature of the interview	1.0 (0.7)	1.1 (0.5)	0.7	NS
Total score	3.9 (1.3)	4.3 (1.1)	2.2	0.042
**Perception**				
Assesses the patient's starting point	1.5 (0.7)	1.8 (0.5)	1.8	NS
Makes it clear that serious/important information is to follow	0.8 (0.5)	1.1 (0.7)	1.9	NS
Uses patient's response to guide the next steps in moving forward	1.5 (0.6)	1.8 (0.4)	2.9	0.009
Total score	3.9 (1.6)	4.6 (1.4)	2.5	0.020
**Invitation**				
Discovers what other information would help them and responds to this	1.2 (0.6)	1.6 (0.5)	2.9	0.008
**Knowledge**				
Gives explanation in an organized manner using “bite-size pieces”	1.4 (0.5)	1.7 (0.5)	3.1	0.005
Uses clear language and avoids jargon and confusing language	1.6 (0.5)	1.8 (0.4)	2.6	0.017
Total score	3.0 (1.0)	3.4 (0.8)	3.6	0.002
**Emotions**				
Picks up and responds to patient's non-verbal cues	1.4 (0.6)	1.5 (0.5)	0.4	NS
Allows patient time to react (use of silence), allows for time to think	1.8 (0.4)	1.9 (0.2)	0.9	NS
Encourages patient to contribute reaction, concerns, and feelings and then responds to him/her	1.6 (0.5)	1.7 (0.4)	1.4	NS
Acknowledges patient's concern and feelings as well as values and accepts legitimacy	1.7 (0.4)	1.8 (0.4)	1.1	NS
Uses empathy to communicate appreciation of the patient's feelings or predicament	1.7 (0.5)	1.8 (0.4)	1.5	NS
Demonstrates appropriate non-verbal behavior	1.8 (0.3)	1.8 (0.3)	0	NS
Provides support	1.7 (0.4)	1.7 (0.4)	0.7	NS
Total score	11.6 (2.4)	12.1 (2.3)	1.6	NS
**Summary**				
Summarizes at the end with a plan to follow up	1.3 (0.4)	1.7 (0.4)	3.6	0.002
Total questionnaire score	24.9 (6.0)	27.8 (5.2)	5.6	<0.001

*Paired t-test assessed change pre-workshop to 2-month post-workshop scores (2, good; 1, adequate; 0, not done/inadequate); NS, non-significant*.

### Resident Self-Assessment

In Table 2 we summarize mean scores of residents' self-confidence in their ability to communicate difficult news before, immediately after, and 2-months after the workshop. Mean questionnaire scores increased from 3.6 to 4.0 (*t* = 7.3, *p* < 0.001) between pre- and post-workshop, reflecting an improvement in residents' confidence. Mean scores remained high after 2 months (mean score 4.2, *t* = 5.2, *p* < 0.001), showing an enduring effect for the workshop. Paired *t*-tests also showed significant change between pre- and post-workshop, and between pre- and 2-month post-workshop for 11 (85%) of the 13 questionnaire items. The mean total score for the 6-item self-report satisfaction questionnaire was 4.4 ± 0.7, indicating high satisfaction with the workshop (see Table 3).

**Table 2:** Residents' self-ratings for confidence in their ability to communicate before, immediately after, and 2 months after the workshop.

	**Pre (*n* = 28)**	**Post (*n* = 28)**	**F/U (*n* = 24)**	**Pre to post**	**Pre to F/U**
**I feel confident in my ability to…**	**Mean (SD)**	**Mean (SD)**	**Mean (SD)**	***p***	***p***
Break bad news in general	3.4 (0.9)	4.0 (0.5)	4.2 (0.6)	<0.001	0.003
Create a supportive environment	3.9 (0.7)	4.2 (0.6)	4.4 (0.7)	0.026	0.004
Reduce or eliminate signs that I am nervous or anxious	3.0 (0.7)	3.8 (0.7)	3.9 (0.8)	<0.001	<0.001
Use language that is non-technical and easily understood	4.0 (0.9)	4.4 (0.6)	4.2 (0.9)	0.005	NS
Adjust the rate and amount of information I provide	3.6 (0.7)	4.1 (0.6)	3.9 (0.7)	<0.001	0.095
Listen to patients' concerns	4.2 (0.6)	4.2 (0.6)	4.6 (0.7)	NS	0.030
Explore a patient's expectations	3.6 (0.7)	4.2 (0.6)	4.2 (0.7)	<0.001	0.016
Empathize with a patient	4.2 (0.7)	4.4 (0.6)	4.6 (0.6)	0.057	0.022
Avoid portraying more hope or optimism that I believe exists to deal with the patients' emotions	3.1 (0.9)	4.0 (0.7)	4.1 (0.7)	<0.001	<0.001
Summarize information in a way that is easy to understand	3.6 (0.6)	4.0 (0.8)	4.1 (0.7)	0.017	0.001
Anticipate possible responses by patients	3.3 (0.8)	3.7 (0.7)	3.8 (0.5)	0.003	0.002
Deal with difficult emotions from families	3.3 (0.9)	3.7 (0.7)	4.0 (0.7)	0.005	0.007
Close the conversation in an appropriate way	3.6 (0.8)	4.0 (0.6)	4.3 (0.6)	0.007	<0.001
Mean score	3.6 (0.4)	4.1 (0.4)	4.2 (0.4)	<0.001	<0.001

*Paired t-test assessed residents' self-report changes from pre- to post-workshop and pre-workshop to 2-month post-workshop; Items ranged from 1 (strongly disagree) to 5 (strongly agree) on a Likert-type scale; NS, non-significant*.

**Table 3:** Residents' self-efficacy and satisfaction ratings at the end of the workshop.

**To what extent do you think…**	**Mean**	**SD**
You will use the tools you learned during this day?	4.6	0.6
You would like to have other learning opportunities with SPs in the future?	4.3	0.9
You learned about yourself as a professional?	4.0	0.9
The discussion with the video contributed to your learning?	4.3	0.9
The discussion was fruitful and productive?	4.4	0.8
The workshop was organized logistically/administratively?	4.7	0.6
Mean score	4.4	0.7

*Workshop participants rated each of the items on a Likert-type scale from 1 (“not at all”) to 5 (“very much”)*.

## Discussion

The purpose of this study was to test the efficacy of a 5-h simulation-enhanced workshop in improving psychiatric residents' self-confidence and communication skills sharing difficult news with patients and their family members. As hypothesized, we found an increase in communication skills as evaluated by external evaluators blind to the timing of simulated interactions (before or 2 months after the training). We also found an increase in residents' self-confidence after attending the workshop, a change that endured 2 months later. Our findings strengthen the existing knowledge (42, 43) on the positive effect of SP-based training in psychiatry, and expand it by demonstrating objective and subjective improvements that persist 2 months after training.

Our simulation-enhanced workshop differs from previous efforts in other areas of medicine. Other workshops have focused mainly on “breaking bad news” (28, 37), whereas we attempted to address a wider spectrum of difficult news, as specifically pertinent to psychiatry. In addition to sharing new diagnoses (schizophrenia or autism), the scenarios and role play vignettes we developed included discussions about involuntary hospitalization, the need for chronic medication use, considerations around medication use during pregnancy and, a driver's license suspension due to a psychotic episode.

Unlike other fields in medicine with more discrete diagnostic criteria, sharing diagnoses in psychiatry can be a continuous and iterative process, often requiring more than one session (6). Sharing information about diagnosis of a serious mental illness is particularly challenging, as the nature of mental illness is often difficult to explain, since there may be no clear etiology and treatment options and prognosis may vary widely. In addition, newly diagnosed individuals with mental health disorders often may not accept their diagnosis due to lack of insight, impaired reality testing or cognition, or stigma related to their condition (44). Moreover, diagnostic information is not the only difficult information that a psychiatrist is likely to share with patients or family members. We addressed this variability through a range of different SP scenarios, discussions, and role plays.

Our main finding was a significant increase in the total mean scores of communication skills at two-months follow-up. This finding is in line with a previous study (41) among 39 pediatric residents, which showed a similar effect in improving residents' communication skills. However, and in contrast to that study, we found that even though residents' self-confidence improved across all self-rated domains, blinded evaluators found no change on items assessing the way the residents addressed the SP's *Emotions*. This finding was inconsistent with other studies showing a change across all SPIKES domains (41, 45). Three possible explanations may address this discrepancy: First, our study assessed a 2-month follow-up interval, compared to an immediate change in other studies. Second, the pre- and post-workshop mean scores were both relatively high for these items, indicating a possible ceiling effect. Third, the lack of change may be attributed to differences between the simulated environment and the residents' daily clinical reality, as some simulated scenarios may not represent the full complexity of real patients.

We have recently described a new model of SP-based learning that can help address the last of these three possibilities. In this model, termed Co-constructive Patient Simulation (CCPS) (46), a designated learner creates a case script based on a challenging encounter faced during clinical practice. Together with an instructor, the learner is then involved in creating, editing, and practicing role play of the simulated case with an SP. After the creation of the simulated environment, fellow learners with no prior knowledge of the case interview the SP. The educational encounter is followed by a group debriefing. We piloted the CCPS model with 11 trainees in child and adolescent psychiatry throughout a full academic year. The topics chosen by the 6 designated learners included: medical errors and error disclosure, racial tensions and overt racism, interprofessional conflicts, transphobia, patient-on-provider violence, and sexual health. The residents, who rated the model highly, shared that they were engaged and could openly discuss emotionally challenging topics. In sum, it seems that for the unique challenges of sharing difficult news in psychiatry, a learner-centered simulation approach may create even more meaningful and relevant learning opportunities.

### Limitations

Our study has several limitations. First, lacking a comparison to other teaching methods or a non-training control group, we conducted within-group pre- and post-workshop tests. Consequently, improvement in communication and clinical skills could be attributed in part to other factors, such as site-specific training opportunities, given that residents continued their training and had other psychiatric learning experiences during the 2-month interval between the pre- and post-workshop assessments. Second, our sample consisted of only 24 residents who completed the study, thus limiting generalizability and statistical power. Third, our study included residents in different training years, introducing heterogeneity to the study sample, even though we found no differences in outcomes between more and less experienced residents. Finally, given the small sample size and exploratory nature of our study, we recognize that our item-level findings would have been more modest had we made corrections for multiple comparisons. For example, in the case of Table 1, the Bonferroni significance threshold would have been a more conservative 0.0038 (i.e., 0.05/13). Nevertheless, an approach of not adjusting for multiple comparisons is preferable as it leads to fewer errors of interpretation when the data under evaluation are not random numbers but actual observations (47).

## Conclusions

This study presented and evaluated the efficacy of a model for teaching psychiatric residents how to better communicate diagnostic and other difficult information. Participants found the simulation-enriched workshop to be useful and demonstrated objective improvements in their abilities to deliver difficult news. The training further enhanced participants' subjective sense of confidence and competence in those communication skills. Sharing diagnoses and other difficult information in psychiatry is challenging, and future studies should focus on developing and investigating ways to facilitate this clinical process.

## Data Availability Statement

The original contributions presented in the study are included in the article/supplementary material. Further inquiries can be directed to the corresponding author/s.

## Ethics Statement

The studies involving human participants were reviewed and approved by the Sheba Medical Center Institutional Review Board approved the study (Protocol #SMC5912-19). The patients/participants provided their written informed consent to participate in this study.

## Author Contributions

DA and AM developed the study design. DA and LK performed the analytic calculations. MM and OS performed the evaluations. RG supervised the project. All authors contributed to the final version of the manuscript.

## Conflict of Interest

The authors declare that the research was conducted in the absence of any commercial or financial relationships that could be construed as a potential conflict of interest.
